# Wheat Ear Recognition Based on RetinaNet and Transfer Learning

**DOI:** 10.3390/s21144845

**Published:** 2021-07-16

**Authors:** Jingbo Li, Changchun Li, Shuaipeng Fei, Chunyan Ma, Weinan Chen, Fan Ding, Yilin Wang, Yacong Li, Jinjin Shi, Zhen Xiao

**Affiliations:** School of Surveying and Mapping Land Information Engineering, Henan Polytechnic University, Jiaozuo 454000, China; lijingbo1024@163.com (J.L.); feishuaipeng@163.com (S.F.); mayan@hpu.edu.cn (C.M.); 211904020046@home.hpu.edu.cn (W.C.); 212004020067@home.hpu.edu.cn (F.D.); 211904010019@home.hpu.edu.cn (Y.W.); 211904020026@home.hpu.edu.cn (Y.L.); 211804010022@home.hpu.edu.cn (J.S.); 212004020068@home.hpu.edu.cn (Z.X.)

**Keywords:** RetinaNet, deep learning, transfer learning, wheat ears, Global WHEAT

## Abstract

The number of wheat ears is an essential indicator for wheat production and yield estimation, but accurately obtaining wheat ears requires expensive manual cost and labor time. Meanwhile, the characteristics of wheat ears provide less information, and the color is consistent with the background, which can be challenging to obtain the number of wheat ears required. In this paper, the performance of Faster regions with convolutional neural networks (Faster R-CNN) and RetinaNet to predict the number of wheat ears for wheat at different growth stages under different conditions is investigated. The results show that using the Global WHEAT dataset for recognition, the RetinaNet method, and the Faster R-CNN method achieve an average accuracy of 0.82 and 0.72, with the RetinaNet method obtaining the highest recognition accuracy. Secondly, using the collected image data for recognition, the *R*^2^ of RetinaNet and Faster R-CNN after transfer learning is 0.9722 and 0.8702, respectively, indicating that the recognition accuracy of the RetinaNet method is higher on different data sets. We also tested wheat ears at both the filling and maturity stages; our proposed method has proven to be very robust (the *R*^2^ is above 90). This study provides technical support and a reference for automatic wheat ear recognition and yield estimation.

## 1. Introduction

Wheat is the largest grain crop in world trade [1,2]. Recently, with population growth and social and economic development, the demand for wheat has increased. However, due to extreme weather, pests, crop diseases, and yield, the wheat supply is unstable [3]. Therefore, maintaining a high and stable wheat yield is essential to improving people’s living standards, maintaining social stability, and promoting the development of the national economy [4]. The number of wheat ears is an important factor that directly affects wheat yield [5,6,7]. Therefore, rapid and accurate identification and statistics of wheat ears are fundamental for crop growth monitoring and yield estimation [8]. Traditional counting methods rely on field surveys, sampling, and weighing. These methods are inefficient, costly, and difficult to determine accurate yield estimation for large areas, severely limiting their application for breeding, monitoring plant performance in crop management, or predicting grain yield. No model has been able to perform consistently across different wheat reproductive stages and identify the derived wheat spikes well. Additionally, some spike counting methods are based on wheat spike data collected at maturity and other traits, which are not suitable for early yield prediction [9]. In recent years, many studies that apply deep learning techniques [10] to unmanned aerial systems [11,12] for wheat spikelet detection under field conditions have received much attention.

In recent years, with the development of artificial intelligence [13], the target detection models built using deep learning, e.g., Faster regions with convolutional neural networks (R-CNN), has far surpassed traditional target detection techniques in feature representation, reaching top performance in terms of detection accuracy and speed [14]. However, deep learning techniques are limited by a large number of training datasets and training equipment. Since the color, shape, and awn of wheat ears change with growth, the same wheat varieties differ in performance in different growing regions. Thus, it is crucial to study a deep learning model for regional wheat detection. The current Faster R-CNN method achieves better detection accuracy in wheat ear detection [15], but its detection speed cannot meet the integration requirements in the unmanned aerial vehicle (UAV) system. Therefore, it is of great significance and application value to develop a wheat detection model with high detection accuracy, migration ability and be integrated into the UAV system.

The target detection models built using deep learning are mainly divided into two categories: two-stage and one-stage. The most representative target detection algorithms for two-stage detectors are Fast R-CNN [16], Faster R-CNN [14], and the most representative target detection algorithms for one-stage detectors are YOLO (You Only Look Once) [17,18] and RetinaNet [19]. Two-stage detectors are usually slower than one-stage detectors. In two-stage detectors, the first step determines the regions (regions of proposals) that might contain a target to be detected (location). The second step performs a detailed identification of the target contained in each candidate region (classification) [14]. RetinaNet combines the advantages of multiple target recognition methods, especially the“anchor” concept introduced by Region Proposal Network (RPN) [14], and the use of feature pyramids in Single Shot Multibox Detector (SSD) [20] and Feature Pyramid Networks (FPN) [21]. Retinanet has a wide range of applications, such as ship detection in remote sensing images of different resolutions [22], identification of storm drains and manholes in urban areas [23], fly identification [24], and rail surface crack detection [25].

The future development trend of the image-based automatic recognition method is to obtain wheat ears and yield data in real-time over a large area. Using machine learning techniques, the image-based recognition method exploits the color, texture, and shape of the target image to encode and represent the image. Thus, image feature representation is essential [26]. However, these features are different in different environmental conditions, limiting the effectiveness of these features. For example, [27] exploited color consistency coefficient, gray symbiosis matrix, and edge histogram to construct wheat ear feature matrix. The authors of [28] used scale-invariant feature transform (SIFT), and Fisher vector (FV) features to identify the wheat ears at the heading stage accurately. The authors of [9] created a binary image through the local maximum value of the pixel and the variance of the nearest neighbor pixel to calculate the number of wheat ears in the image. The experimental results of these studies may suffer from degradation due to different recognition angles of the wheat ears being photographed, the period of growth of the ears, and the field environment in which the wheat ears were photographed. As a branch of machine learning, current deep learning techniques can solve this problem for wheat recognition. Deep Learning [29] exploits a perceptron containing multiple hidden layers, and it transforms the features of samples from the original space to a new feature space based on the principle of learning hierarchical data. In this process, the hierarchical feature representation was automatically learned and obtained, and the accuracy of recognition improved [29]. The main advantage of deep learning techniques is that the characteristics of the input data are automatically learned, overcoming the bottleneck in many intelligent applications. Thus, the use of deep learning techniques has become the frontier in the field of crop phenotypes.

The current application of drones combined with deep learning technology has greatly promoted the development of precision agriculture. In recent years, some meaningful research [7,8,9,15,27,28,30,31,32,33,34,35,36,37,38,39,40,41,42] has emerged. These studies have used RGB (red, green, blue), multispectral, hyperspectral, and thermal infrared data acquired by UAV and CNN to evaluate the phenotypic characteristics of citrus crops [38], obtain key points of plants/plant leaves [39], plant stress analysis and plant disease identification [40,41]. The research on automatic recognition and counting wheat ears with deep learning technology has made great progress [8,15,37]. Hasan et al. [8] used R-CNN to identify and count wheat ears. Madec et al. [15] used Faster R-CNN to identify wheat ears in RGB images with different spatial resolutions. Sadeghi–Tehran et al. [37] proposed a visual recognition method based on linear iterative clustering and deep CNN to automatically identify and count wheat ears in the images obtained under natural field conditions. The research works [8,15,37] achieved a lower detection speed than [30,42], and cannot be integrated into UAV systems for real-time detection.

To our knowledge, there are no other systematic, quantitative assessments of how training sample size and sample selection methods affect the results of wheat identification models. This paper aims to: (1) Obtain a model with high recognition accuracy at different growth stages; (2) evaluate the impact of training samples on Faster-RCNN and RetinaNet in combination with transfer learning; (3) evaluate the detection speed of Faster-RCNN and RetinaNet. To achieve these goals, there are many different types of wheat ears in different growth environments considered in this paper, and a model suitable for regional wheat identification is proposed. The idea of transfer learning is integrated into the proposed model to explore its performance in different training samples, wheat fertility training samples, and obtain the recognition performance of the model in wheat ear images. Specifically, images of wheat ears in different fertility periods collected in the field are used in combination with the wheat ears recognition image database (Global WHEAT). Different deep learning models based on different data samples of wheat ears in different fertility periods are trained by labeling wheat ears. The recognition accuracy and detection speed of these models are then compared and analyzed. The model proposed in this paper achieves high detection accuracy and migration capability, and it can be integrated into UAV systems.

## 2. Materials and Methods

### 2.1. Data Acquisition and Processing

#### 2.1.1. Global Wheat Data Acquisition

Global Wheat Head Detection (Global WHEAT) [43] obtains data from the wheat ear recognition image database (data source: https://www.kaggle.com/c/global-wheat-detection/data, accessed on 12 June 2020). The database was carefully produced by seven countries, nine research institutes, and more than ten phenotyping experts in a year. This database is composed of train.zip, test.zip, sample_ssubmission.csv, train.csv, and other files. The information contained in each file is listed in Table 1.

**Figure 1:**
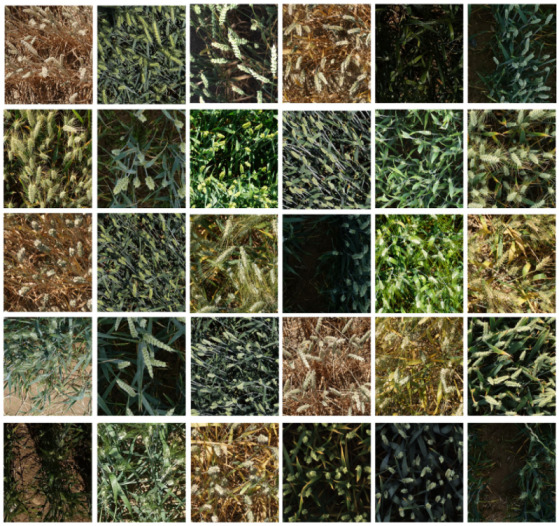
Part of the image data in the Global WHEAT database.

The Global WHEAT data set contains a total of 3422 images, and each image has a size of 1024 × 1024 pixels. The Ground Sampling Distance (GSD) of the GWHD dataset ranges from 0.28 to 0.55 mm [43]. Part of the image data is shown in Figure 1.

#### 2.1.2. Digital Image Data Acquisition

**Figure 2:**
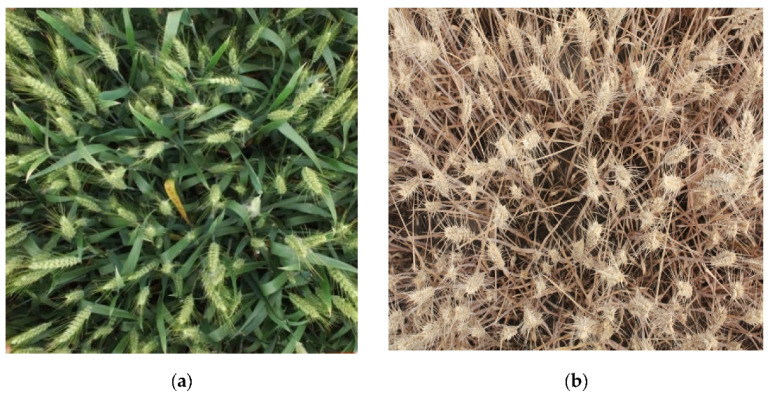
Digital images of wheat in key growth stages. (**a**) Partial Filling stage; (**b**) Mature stage.

The digital image of wheat in the filling stage and mature stage are obtained by a high-definition digital camera. In clear and windless conditions, the backlit hand-held Sony DSC-H9 digital camera was used for vertical shooting. The shooting height was approximately 1 m higher than the top of the wheat canopy, and the shooting area was approximately 0.75 M^2^ (5 rows of wheat with a spacing of 15 cm). Each digital image has 3088 × 2056 pixels, and the horizontal and vertical resolution is 72 dpi. A total of 715 images are taken. Among these 715 images, 365 images present the wheat ears in the filling stage and 350 in the mature stage with an approximate ratio of 1:1. The digital images of wheat ears in the partial filling stage and mature stage are illustrated in Figure 2.

According to the number of images, three groups of digital images of wheat in the filling and mature stages are set as the training data set, and the number of images in each group is 50, 100, and 150, respectively. The denotations of the three situations are listed in Table 2, where Filling Stage Model (FSM) and Mature Stage Model (MSM) represent a wheat data set at the grain filling stage and mature stage.

According to the number of wheat ears in each image, the test data set is divided into three groups, and the number of images in each group is 30. The detailed information of the training data set and the test data set is listed in Table 2 and Table 3. Testing the same number of images ensures that each model has the same evaluation benchmark, and the test set is 180 images in total. The ratio of 90 images for each fertility period is 1:1, which guarantees the reliability of the results.

#### 2.1.3. Data Processing

(1) Image marking

To obtain better recognition results, many deep learning models require annotated training data sets. Although image-based high-throughput crop phenotyping systems already exist, such as Field Scanalyzer [44], which generates a large amount of image data every day, the annotated images with ground truth values are not available among the obtained crop image data. Therefore, the obtained images must be labeled to generate a training data set.

**Figure 3:**
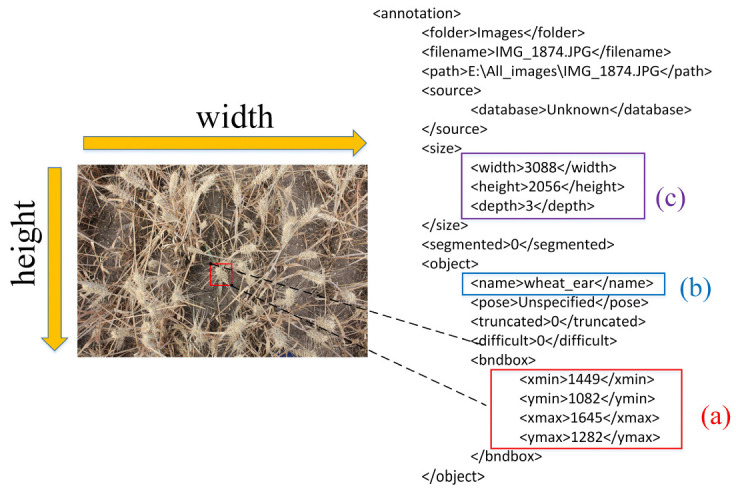
Wheat ear label image data. (**a**) the position of wheat ears in the image; (**b**) the name of the target; (**c**) the shape of the image.

LABELIMG [45] is a free and open-source graphic image annotation tool (https://github.com/tzutalin/labelImg, accessed on 13 June 2020) that grants simultaneous access to different users and is available to all institutions. The LABELIMG tool outputs an annotation file with an interactive drawing of a bounding box containing all the pixels of the wheat ears. After the digital image is obtained, theannotation tool of LABELIMG can be used to draw bounding boxes around each identified wheat ear in the images. Specific information for each image is shown in Figure 3; Figure 3a exhibits the position of wheat ears in the image; Figure 3b illustrates the name of the target, and Figure 3c indicates the shape of the image. The bounding boxes contain all the pixels of the wheat ears, however, sometimes the bounding box can be too large and includes background lawns. If possible, the boxes also contain a small portion of the wheat stem. When the identification results from one of the developed models were compared, it was found that a few wheat ears were forgotten by the operator during the interactive labeling process. Therefore, the images were reprocessed with greater care.

Each labeled image has an additional text file containing the coordinates of the annotated bounding boxes. In this file, the boxes are stored as a 4-tuple (x_min_, y_min_, x_max_, y_max_), where (x_min_, y_min_) and (x_max_, y_max_) denote the top left corner and the lower right corner of the box, respectively.

(2) Denoising and enhancement

During the shooting process, the wheat image is easily affected by the changes in natural light, growth environment, shaking of the shooting equipment, and the unstable focus of the lens. Meanwhile, the obtained image may contain some noise caused by random signals in the process of transmission [34]. Therefore, the method of data denoising is exploited to remove the noise points in the obtained image and reduce the influence of noise on the recognition results. Firstly, the median filtering method with a kernel of 5 is used to remove the noise in the wheat image. The specific denoising process exploits the Python language to call the medianBlur function provided by the OpenCV library, and the parameter ksize is set to 5 [34].

**Figure 4:**
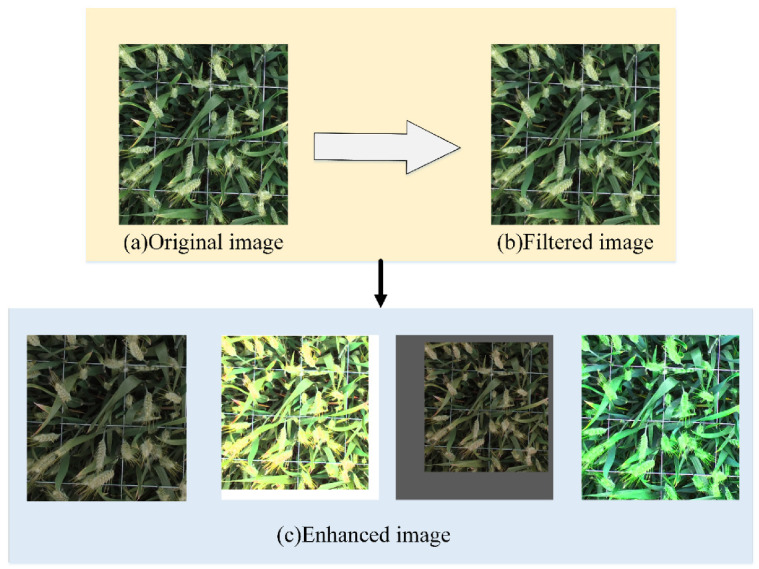
Image denoising and data enhancement. (**a**) original image; (**b**) filtered image; (**c**) enhanced image.

In the training process, the training samples cannot always reflect all the information for each real target. Thus, the image data is enhanced by different transformations to improve the training data and generalization ability. The diversity allows the model to be applied to various situations and has more robustness. Additionally, most deep learning algorithms require a large amount of training data to obtain accurate recognition results. Although this work obtained approximately 3000 images, there were not enough to train and validate the model, emphasizing the need for data enhancement. In order to address this issue, programs have been written in Python language to shrink, enlarge, and flip the original image [34]. In order to simulate the change of light, the HSV (hue, saturation, and value) [46], color space, and various conversions are exploited, such as linear change of hue, linear change of saturation, and linear change of brightness. PIL (Python Image Library) [47] is a third-party image processing library provided by the python (https://python-pillow.org/, accessed on 10 September 2020) language. This library is featured with extensive file format support, efficient internal representation, and strong image processing capabilities. It provides a solid foundation for general image processing tools. The function FLIP_LEFT_RIGHT can flip an image horizontally. Specifically, a Python program is written to call the transpose function provided by the PIL library, and the parameter of FLIP_LEFT_RIGHT is used. Based on this, each HSV channel’s value is changed linearly and randomly; the rand function provided by the Numpy library randomly returns a value between 0 and 1 for multiplication. The image denoising and data enhancement effects are illustrated in Figure 4.

### 2.2. Method

The application of deep learning techniques has greatly contributed to the development of precision agriculture. Faster R-CNN has been widely applied and used in maize tassels detection [35] and wheat ears recognition [15]. RetinaNet combines the advantages of multiple target recognition methods, especially the “anchor” concept introduced by Region Proposal Network (RPN) [14], and the use of feature pyramids in Single Shot Multibox Detector (SSD) [20] and Feature Pyramid Networks (FPN) [21]. Retinanet has a wide range of applications, such as ship detection in remote sensing images of different resolutions [22], identification of storm drains and manholes in urban areas [23], fly identification [24], and rail surface crack detection [25]. The experiment described in this paper was conducted on a computer equipped with Intel^®^ Xen(R) W-2145 CPU and NVIDIA GeForceRTX 2080Ti. The Keras library based on the Tensorflow environment in Windows was employed. Additionally, the Python language was employed to realize the automatic recognition of wheat ears based on Faster R-CNN and RetinaNet and verify the recognition accuracy.

#### 2.2.1. Faster R-CNN

As a typical two-stage target recognition algorithm, Faster regions with convolutional neural networks (Faster R-CNN) [14] has been widely applied to many fields since it was proposed. Faster R-CNN is an improved version of Fast-RCNN [16], which uses RPN network (Region Proposal Network) instead of Selective Search to generate candidate boxes. Additionally, the anchor concept is introduced and can be used in future target recognition models.

**Figure 5:**
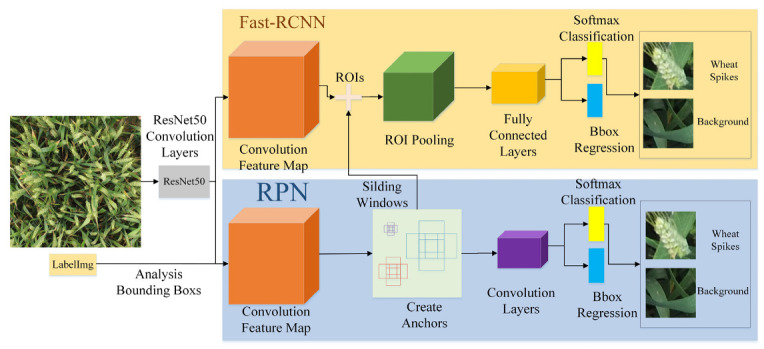
The diagram of the Faster R-CNN framework.

As shown in Figure 5, Faster R-CNN consists of four parts:

(1) Convolution layer.

As a CNN network for target recognition, the convolution layer in Faster R-CNN uses ResNet50 as the feature extraction network. The network in this layer extracts the feature map of the image, which is passed to the subsequent RPN layer and the fully connected layer;

(2) RPN layer.

This layer is used to generate target candidate regions, eliminating the time consumed by the process of Selective Search (pre-SS) [48] that generates candidate frames. Faster R-CNN uses an RPN network that shares part of the weight with the recognizer to generate candidate frames for the image directly, and then perform classification and position regression based on the candidate frames obtained by RPN;

(3) Region of Interest (ROI) pooling layer [14].

This layer uses the feature map and suggestion box information output by the RPN layer to map to feature maps of the same size;

(4) Recognition.

The feature map of the candidate target area is used to calculate the category of the candidate target area, and the coordinate frame position of the target is regressed again to obtain the final precise position of the target [49]. The score threshold of 0.5 was used to determine whether the bounding box contains wheat ears. In order to limit the overlap between bounding boxes containing the same wheat ear, the Intersection-over-Union (IOU) threshold was set to 0.5 so that only one bounding box was selected [28].

#### 2.2.2. RetinaNet

**Figure 6:**
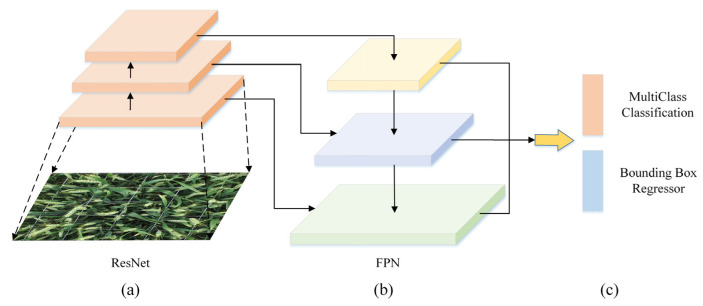
The structure of RetinaNet. (**a**) Backbone network; (**b**) decoder; (**c**) subnet).

Retinanet combines the advantages of multiple target recognition methods, especially the “anchor” concept introduced by RPN and the use of feature pyramids in Single Shot Multibox Detector (SSD) [20] and Feature Pyramid Networks (FPN) [21]. The structure of RetinaNet is composed of three parts: a convolutional neural network for feature extraction and two sub-networks for classification and box regression [19]. The structure is shown in Figure 6, where Figure 6a represents the backbone network, i.e., ResNet50; Figure 6b illustrates that FPN is used as a decoder to generate a multi-scale convolutional feature pyramid, and Figure 6c shows that two subnets are used for classification and bounding box regression. Based on feature mapping, two sub-networks of classification and box regression are constructed through simple convolution operations. Specifically, the classification sub-network performs object classification, and the box regression sub-network is used to return the position of the bounding box. The advantage of FPN is that the hierarchical structure of the deep convolutional network can be used to represent multi-scale objects to help the recognizer create a better prediction of the position.

This paper uses ResNet50 to extract image features [50]. Compared with the two-stage recognition method, the low accuracy of the one-stage target recognition is mainly caused by the extreme imbalance between the foreground and the background during the training process of the dense recognizer, which creates a large number of negative samples during the training process [19]. The focus loss is used to solve the problem of extreme imbalance of categories; it is implemented by modifying the standard cross-entropy, reducing the loss assigned to well-classified examples [19]. Under the supervision of focus loss, the retina can achieve significant improvements on the universal object recognition benchmark. It is expressed as Equation (1) and has been used to improve detection accuracy [19]. The definition of an a-balanced variant of the focus loss is:

(1) $$FL(p_{t})=-\alpha_{t}{\left(1-p_{t}\right)}^{y}\log(p_{t})$$

where α*_t_* and *γ* are hypermeters. $\alpha_{t}\in \left[0,1\right]$ is the weighting factor to address class imbalance; parameter *γ* smoothly adjusts the rate at which easy examples are down-weighted [19]. For a convenient notation, $p_{t}$ is defined as follows.

(2) $$p_{t}=\left\{ \begin{array}{l} p\;\;if\;y=1\\ 1-p\;\;otherwise \end{array}\right.$$

where p∈[0,1] is the probability estimated by the model, and *y* = 1 specifies the ground truth.

#### 2.2.3. Recognition Accuracy Evaluation Index

The accuracy of wheat ear recognition is evaluated by precision and recall [51,52]. Precision measures the accuracy of the algorithm, recall measures the integrity of recognition, and *F*1-*score* is used to balance precision and recall. The classifier with a high *F*-*score* is now shown to have good recall and accuracy. The three indicators can be calculated as follows:

(3) $$Precision=\frac{TP}{FP+TP}$$

(4) $$Recall=\frac{TP}{FN+TP}$$

(5) $$F1-score=2\times \frac{Precision\times Recall}{Precision+Recall}\times 100\%$$

If the predicted bounding box overlaps with the marked ear bounding box and exceeds the IOU threshold (set to 0.5 in this paper), then the predicted bounding box represents the wheat ear sample; otherwise, it is the background sample. *TP* indicates the number of correctly classified wheat ear samples, and *FN* indicates the number of wrongly classified wheat ear samples. *FP* indicates the number of background samples that are wrongly classified, and TN indicates the number of background samples that are correctly classified.

Average Precision (*AP*) balances the precision and recall values, reflecting the model’s performance [53]. Considering the accuracy as the ordinate and the recall as the abscissa, a Precision and Recall (*PR*) curve can be obtained; the area under the curve is *AP*.

(6) $$AP=\int_{0}^{1}P(R)$$

Additionally, indicators such as the mean absolute error (*MAE*), the root mean squared error (*MSE*), the relative *RMSE* (*rRMSE*), bias (*BIAS*), and coefficient of determination (*R*^2^) are used to evaluate the result of wheat ear recognition. *MAE* and *rRMSE* represent the accuracy of recognition, and *MSE* represents the robustness of the recognition model. The lower the scores of *RMSE*, *rRMSE*, and *MAE*, the better the performance of the model. These indicators can be calculated as follows:

(7) $$RMSE=\sqrt{\frac{1}{N}\sum_{k=1}^{n}{\left(Truth_{k}-Predicted_{k}\right)}^{2}}$$

(8) $$rRMSE=\sqrt{\frac{1}{N}\sum_{k=1}^{n}{\left(\frac{Truth_{k}-Predicted_{k}}{Truth_{k}}\right)}^{2}}$$

(9) $$BIAS=\frac{1}{N}\sum_{k=1}^{n}\left(Truth_{k}-Predicted_{k}\right)$$

(10) $$MAE=\frac{1}{N}\sum_{k=1}^{n}\left|Truth_{k}-Predicted_{k}\right|$$

(11) $$R^{2}=1-\frac{\sum_{k=1}^{n}{\left(Truth_{k}-Predicted_{k}\right)}^{2}}{\sum_{k=1}^{n}{\left(Truth_{k}-{\bar{Truth}}_{k}\right)}^{2}}$$

In Equations (7)–(10), *N* represents the number of test images for the model, the actual number of wheat ears, the number of identified wheat ears of the *k*-th image, and the average actual number of wheat ears, respectively.

## 3. Results

### 3.1. Analysis of the Recognition Results Obtained by Different Methods on the Global WHEAT Dataset

**Figure 7:**
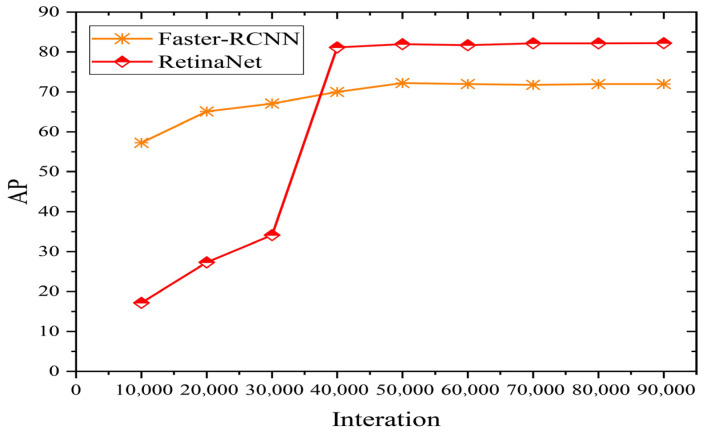
The AP value of Faster R-CNN and RetinaNet for identifying wheat ears.

In order to evaluate the performance of the method used to identify wheat ears in this paper, two target recognition algorithms, Faster R-CNN and RetinaNet, as shown in Figure 5 and Figure 6, are used. These three models are trained on the same data set (Global WHEAT data set), and the mean average precision (mAP) results of the test data set are shown in Figure 7. Since the wheat ear is the only identification target, mAP is equal to average precision (*AP*).

A total of 90,000 iterations were executed to train the model, where 10,000 iterations were executed to calculate the AP value of the Faster R-CNN and RetinaNet (IOU set to 0.5). Each model uses the VOC data set for pre-training and initialization. The AP value and loss of Faster R-CNN and RetinaNet for identifying wheat ears are shown in Figure 7. As the number of iterations increases, the accuracy of the model gradually increases. When the number of iterations reaches 40,000, the accuracy of the model reaches its maximum. The Faster R-CNN model achieves high accuracy from the beginning, and the AP value does not increase significantly as the number of iterations increases. The AP value of the RetinaNet increases significantly between 30,000 to 40,000 iterations and then becomes stable to the maximum. This result is related to the two-part detection of Faster R-CNN. In Faster R-CNN, the first step generates region proposals that may contain a target to be localized, and the second step performs a fine distinction between the specific targets contained in each candidate region. Therefore, high accuracy is achieved at the beginning of the iterative process. By contrast, RetinaNet performs one-stage detection, and it directly generates the position and category information of the target that derived from the object. This method is prone to category classification errors and inaccurate target location information at the beginning. Through continuous iterative training, the focal loss mechanism of RetinaNet continuously and rapidly reduces the loss value, and a stable detection result can be obtained after 35,000 iterations. Additionally, it can be seen from Figure 7 that the AP value of the RetinaNet is higher than that of the Faster R-CNN, indicating that the RetinaNet achieves the best AP; this is because RetinaNet extracts multi-scale semantic features, which greatly improve the AP value. The RetinaNet model has a strong advantage in wheat ear classification and box regression using derivatives.

**Figure 8:**
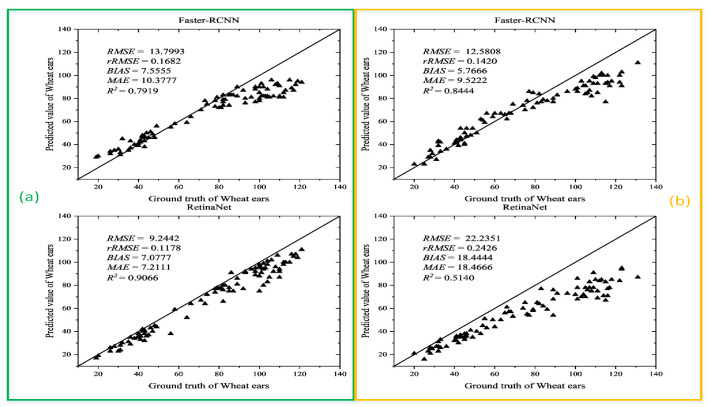
Wheat ear recognition accuracy of Faster R-CNN and RetinaNet models on the test set with images collected in (**a**) in filling stage; (**b**) in mature stage.

Though the Global WHEAT data set contains many types of wheat ear data, there are many wheat varieties globally, and even the same wheat varieties show great differences due to different growth environments. To better evaluate the performance of the Faster R-CNN and RetinaNet models trained on the Global WHEAT data set, the wheat images collected in the field during the grain filling phase and the mature stage were used as the test set. Using the number of wheat ears identified by different models and obtained by manual methods to calculate the RMSE, rRMSE, MAE, Bias, and *R*^2^, the results are shown in Figure 8.

**Figure 9:**
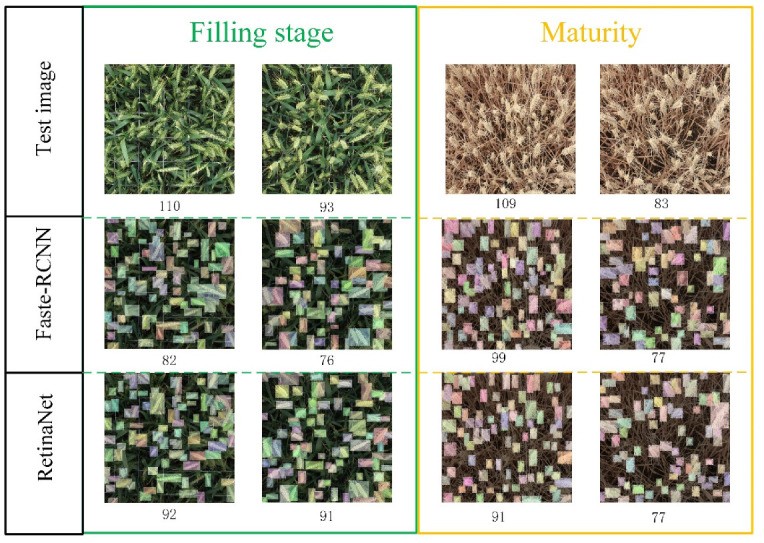
The predicted number of wheat ears (partial) by the Faster R-CNN and RetinaNet models on the test data set.

The results in Figure 8 show that $R^{2}$ of the Faster R-CNN and RetinaNet models on the test data set with images collected in the filling stage are 0.792 and 0.907, respectively. The $R^{2}$ of the RetinaNet is 14.5% higher than that of the Faster R-CNN, indicating that the RetinaNet model achieves the highest recognition accuracy for wheat ears in the filling stage. As for recognizing the wheat ear in the mature stage, the Faster R-CNN model achieves the $R^{2}$ of 0.844, and the RetinaNet model achieves the $R^{2}$ of 0.514. The number under each image represents the number of identified wheat ears, showing that the predicted value differs from the true value by more than 10 wheat ears. Figure 8 and Figure 9 indicate that Faster R-CNN and RetinaNet models trained on the Global WHEAT dataset do not transfer well to the field for wheat ears identification. According to [43], most of the images fromthe Global WHEAT dataset are acquired before the appearance of head senescence. It also demonstrates the limitations of the Global WHEAT dataset. Therefore, it is important to obtain a model that can migrate to the field and perform with high accuracy for wheat ears of different fertility stages.

### 3.2. Results and Analysis of Wheat Ear Recognition Based on Transfer Learning

#### 3.2.1. Recognition Results and Analysis of Different Numbers of Training Samples after Transfer Learning

**Figure 10:**
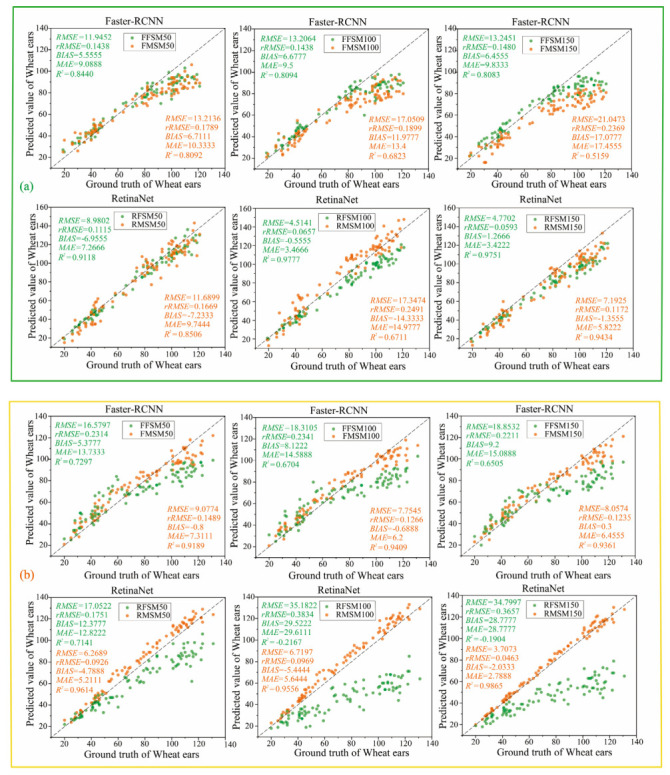
The performance of the models after transfer learning on the test data set with images collected in (**a**) filling stage; (**b**) mature stage.

In order to study the influence of training samples on the model’s performance in the process of transfer learning, the Faster R-CNN and RetinaNet models trained on the Global WHEAT dataset were used as the initial models for transfer learning. Each model was trained 90,000 times. The samples in the test set were used to verify the accuracy of these models, and the results are shown in Figure 10. In this figure, FFSM indicates that the Faster R-CNN model is trained on wheat images collected in the filling stage, and FMSM indicates that the Faster R-CNN model is trained on wheat images collected in the mature stage. Similarly, RFSM and RMSM represent the training of the RetinaNet model on wheat images collected in the filling stage and the mature stage, respectively. Meanwhile, 50, 100, and 150, respectively, represent the number of training sample images.

For different numbers of training samples, manual counting and recognition results have a strong positive correlation. The comprehensive analysis of Figure 8 and Figure 10 indicates that the recognition capabilities of Faster R-CNN and RetinaNet models have greatly improved by transfer learning, and the accuracy of the RetinaNet for recognizing the wheat ear in the filling stage and the mature stage is 97.77% and 98.65%, respectively. Meanwhile, the highest accuracy of the Faster R-CNN model for recognizing the wheat ear in the filling stage and the mature stage is 94.09% and 84.40%, respectively. Therefore, the use of the transfer learning method to place the wheat ear images collected in the field for model training can improve the recognition performance of the model. According to the overall analysis results shown in Figure 10, after 50 training samples were used for training, the recognition performance of RetinaNet and Faster R-CNN models greatly improved compared with the initial model. However, as the number of training samples increased, the recognition accuracy o the two models slowly improved, especially the accuracy of the Faster R-CNN model for recognizing that the wheat in the filling stage was slowly decreasing.

#### 3.2.2. Recognition Results and Analysis of Transfer Learning in Different Growth Stages

**Figure 11:**
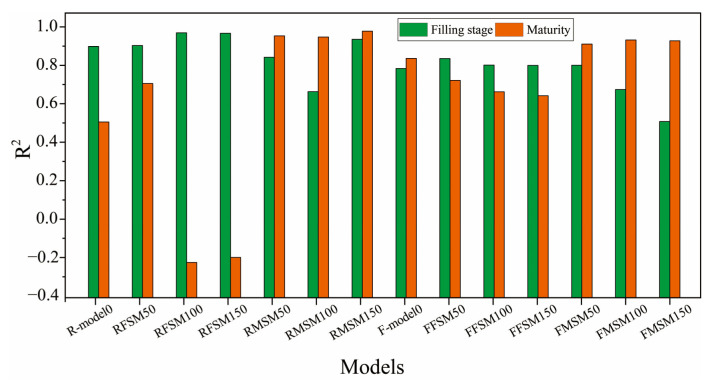
The accuracy of wheat ear recognition by different models in different growth stages.

The deep learning model consumes a lot of time and equipment (such as GPU) for model training. Therefore, a recognition model with high identification accuracy for wheat ears at different fertility stages is essential for crop yield estimation and a better understanding of the wheat ears and canopy. This paper is based on transfer learning to study the recognition performance of the Faster R-CNN and RetinaNet models in different growth stages of wheat ears. For the filling state and the mature state, the *R*^2^ of the Faster R-CNN and RetinaNet models are calculated and the results are shown in Figure 11.

R-model0 and F-model0 in Figure 11 are two initial models trained on the Global WHEAT dataset. It can be seen from Figure 11 that the Faster R-CNN and RetinaNet models trained on images of wheat ears in a specific growth stage obtain good results for identifying wheat ears in the same growth stage. For identifying wheat ears in other growth stages, the recognition performance of the two models decreases; this is mainly due to the change in the color and awns of wheat ears, which result in changes to wheat features extracted by Faster R-CNN and RetinaNet and the corresponding decline in the performance of wheat ear recognition.

Meanwhile, it was found that the RMSM150 model achieves high recognition accuracy for identifying wheat ears at different growth stages (*R*^2^ = 0.9434 for filling stage and *R*^2^ = 0.9865 for mature stage), indicating that the wheat ears in the mature stage exhibit better characteristics for recognition. Thus, the RetinaNet model achieves more robustness in recognition performance. Although RFSM achieves a higher accuracy for recognizing wheat ears, it does not perform as stable as the FFSM model on the test data set with wheat ears in different growth stages. In order to have the RetinaNet model achieve higher recognition accuracy for wheat ears in different growth stages, the combination of images collected for wheat ears in different growth stages can be used in the future.

### 3.3. The Recognition Results and Analysis of RetinaNet

A comprehensive analysis of the experiments presented above indicates that the recognition accuracy of the RetinaNet model is better than that of the Faster R-CNN model. However, these experiments only focus on the wheat ears in a single growth stage and do not consider the wheat ears of multiple growth stages. Therefore, the images of wheat ears in different growth stages can be used as the training data set to study recognition performance of RetinaNet and Faster R-CNN models. Firstly, 150 images of wheat ears in the filling stage and 150 images of wheat ears in the mature stage were used for training. The 300 images contain 39,407 wheat ears in total. The images of the same wheat variety and similar shooting environment are used as test samples. As listed in Table 3, there are 180 images in total, including 13,513 wheat ears.

**Figure 12:**
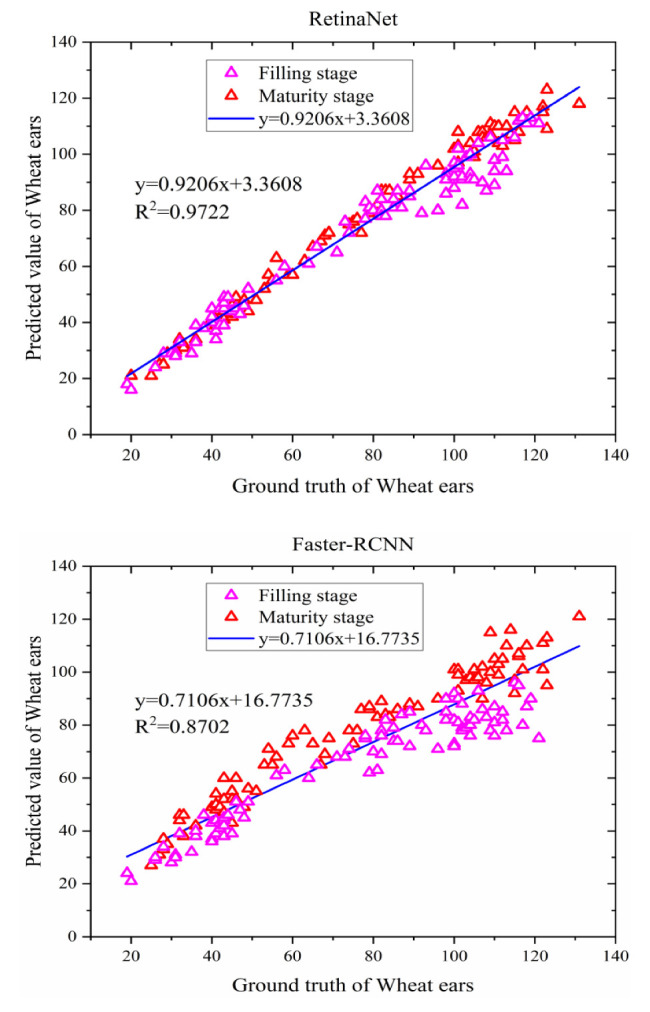
Scatter plot of recognition value and real value of wheat ear in RetinaNet and Faster R-CNN models.

As for the number of wheat ears in 180 images, the relationship between the true value and the recognition value obtained by RetinaNet and Faster R-CNN models is illustrated in Figure 12. For RetinaNet, the slope of the linear equation between the true value and the identification value is 0.9206. The intercept of the linear equation is 3.3608, and it is approximate to 1, indicating that the use of the RetinaNet to identify wheat ears can obtain a result in good agreement with the ground truth value (only 3.36 wheat ear errors, *R*^2^ = 0.9722). Thus, the model can be used for wheat ear identification. Compared with the RetinaNet model, the slope of the linear equation between the recognition value and the true value of the Faster R-CNN model is 0.7106, and the intercept is 16.7735, with *R*^2^ = 0.8702. It can be concluded from the above results that the RetinaNet method is more suitable to identify wheat ears.

It can be seen from Table 4 that the F1-score of RetinaNet improved by 8.92% compared with Faster R-CNN. In addition, based on the same Keras framework and operating environment, the running time of different recognition methods was measured and used to calculate the time needed to recognize the wheat ears in 180 images and calculate the average time needed to recognize wheat ears in a single image. The results indicate that the average time of the Faster-RCNN and RetinaNet is 9.19 and 6.51 s, respectively. The RetinaNet method proposed in this paper can meet the requirements of high recognition accuracy and recognition speed.

## 4. Discussion

Earlier studies [27,28] on wheat recognition are limited by the wheat dataset, which causes the studied wheat recognition models not to be well-migrated for application to other regions. Transfer learning [54] transfers the characteristic information of the target in the source domain to the target recognition, which can greatly solve the problem of lack of data in the target domain. The deep learning method needs a large amount of data to train a model and achieve excellent performance, but data in the field for wheat ear recognition is difficult to obtain and costly to label. To our knowledge, there are no other systematic, quantitative assessments of how training sample size and sample selection methods affect the results of wheat identification models. This paper combines deep learning and transfer learning to study a wheat crop detection model with high accuracy and capable of migration. This technique can be extended to any crop identification.

The method proposed in this paper for recognizing wheat ears based on RetinaNet is compared with the method based on Faster R-CNN proposed by Madec et al. [15]. The RetinaNet method achieves the best recognition performance (AP exceeds 82%) by using the Global WHEAT database [43] as the training set; this shows that the RetinaNet method can be applied to most countries in the world, and appliesto the identification of wheat ears under different wheat distribution densities, different wheat varieties, and different growth environments.

Meanwhile, the RetinaNet and Faster R-CNN models trained on the Global WHEAT data set are applied to the field-collected wheat data based on transfer learning. Compared with the Faster R-CNN method, the recognition performance of the RetinaNet is greatly improved, and the RetinaNet performs the best for recognizing wheat ears in different growth stages; this also indicates that the RetinaNet model has stronger transfer learning ability and better wheat ear recognition performance. These abilities are due to how RetinaNet exploits FPN to extract low-level high-resolution and high-level low-resolution semantics and then uses horizontal connections to combine the corresponding feature maps with reconstructed layers, helping the recognizer better predict the position. Therefore, RetinaNet is more sensitive to the target and achieves excellent recognition performance with transfer learning. Meanwhile, ResNet50 acts as a feature extraction network for both RetinaNet and Faster R-CNN. By adopting a feature pyramid with multi-size feature extraction and output, RetinaNet has an advantage in small target detection such as wheat ears, whereas Faster R-CNN only exploits the last layer of features of the underlying network. Therefore, compared with Faster R-CNN, RetinaNet performs better and is more suitable for wheat ear detection. Since ResNet50 is used as a feature extraction network for RetinaNet, the feature extraction network can also be optimized further to improve the accuracy of wheat recognition in the future.

Additionally, the recognition effect of RetinaNet and Faster R-CNN methods for wheat ears in different growth stages is analyzed. The analysis results show that the *R*^2^ of recognizing wheat ears in the filling stage by RetinaNet and Faster R-CNN methods are 0.978 and 0.844, respectively. Meanwhile, the *R*^2^ of recognizing the wheat ears in the mature stage by RetinaNet and Faster R-CNN methods are 0.986 and 0.941, respectively. It can be seen that RetinaNet achieves the best recognition effect in the mature stage; this is mainly because the characteristics of wheat change with the growth stage. When the wheat grows to maturity, the shape and awns of wheat ears tend to be stable, and the contrast between the wheat ears and leaf background is enhanced, causing difficulty in identifying wheat ears; this is consistent with the conclusions of Hasan et al. [8], Madec et al. [15], and Zhu et al. [28]. Simultaneously, using the RetinaNet method, the accuracy for recognizing wheat ears in the filling stage is only 0.8% lower than that in the mature stage, which also indicates that RetinaNet has a better recognition effect. However, the causes of performance differences between the RetinaNet method and Faster R-CNN for recognizing wheat ears in different growth stages are currently unknown, which will be explored in our future work.

The recognition performance of RetinaNet and Faster R-CNN models trained with different fertility data was analyzed. It can be found in Figure 11 that the accuracy of RetinaNet and Faster R-CNN increased with the number of training data samples. It can also be found that RFSM50, RFSM100, and RFSM150 obtain good recognition results for wheat in the filling stage, but the recognition results for wheat ears in the maturity stage are degraded. The first reason for this is that the color, shape, and awn of the wheat ears change significantly as the wheat grows, and the RetinaNet model trained only with the data of the filling stage fails to detect the mature wheat ears well. It demonstrates the relevance of training samples to the performance of the model, consistent with the current studies [55,56]. Furthermore, it is found that the RMSM50, RMSM100, and RMSM150 models trained only with the data of wheat maturity stage perform the best in detecting wheat ears in the maturity stage; they also achieve better recognition accuracy for the wheat ears in the filling stage. Thus, adding the training of wheat ears at maturity can cause the RetinaNet model to obtain higher recognition performance for both filling and maturity stages. This finding can help transfer the RetinaNet method to wheat ears recognition in other growth regions and different growth stages of wheat and can be useful for studying ear detection models for small samples.

Additionally, the recognition speed of the RetinaNet and Faster R-CNN methods is analyzed in this paper. Under the same framework and operating environment, the average time consumption of the two methods is 9.19 and 6.51 s, respectively. It can be seen that the recognition speed of the RetinaNet method is relatively fast, which is mainly due to the time-consuming extraction of candidate frames in the second stage of the Faster R-CNN network.

The comprehensive analysis of the recognition effect and recognition speed of the two methods indicates that the RetinaNet method is more suitable for wheat ear recognition.

## 5. Conclusions

This paper studies the application of deep learning technology to wheat ear recognition and chooses a better recognition model for recognizing what ears are in different growth stages and the number of wheat ears in a single image. Additionally, the Global WHEAT data set containing images of wheat from different growing environments and varieties are used to generalize training data to create a more robust model. Moreover, the model is integrated with transfer learning to study the transfer ability and recognition performance of the Faster R-CNN and RetinaNet. The comprehensive analysis of the experimental results indicates that the proposed RetinaNet achieves both high recognition performance and recognition speed, which can better meet the requirements of real applications. In our future work we aim to investigate wheat ear recognition on images obtained by unmanned aerial vehicles, which provides a new approach for wheat ear recognition and yield estimation. In order to help researchers reproduce the proposed method, the program file used in our study is provided (https://github.com/lijignbo1024/Program.git, accessed on 8 June 2021).

## Figures and Tables

**Table 1:** The information of the data files in the Global WHEAT database.

Files	Information
train.csv	the training data
sample_submission.csv	a sample submission file in the correct format
train.zip	training images
test.zip	test images

**Table 2:** Training database.

Growth Period	Database	Number of Images Per Group	Number of Wheat Ears Per Piece
Filling stage	FSM50	50	6409
	FSM100	100	12,733
	FSM150	150	19,275
Mature stage	MSM50	50	6684
	MSM100	100	13,404
	MSM150	150	20,132

**Table 3:** Testing database.

Growth Period	Number of Wheat Ears in Each Image	Number of Images Per Group	Number of Wheat Ears Per Piece	Total Number of Wheat Ears Per Piece
Filling stage	less than 50	30	1125	6814
	50–100	30	2458	
	more than 100	30	3231	
Mature stage	less than 50	30	1128	6699
	50–100	30	2116	
	more than 100	30	3455	

**Table 4:** Using different methods for wheat ear recognition.

Methods	F1-Score (%)	Times (s)
Faster R-CNN	82.25	9.19
RetinaNet	91.17	6.51

## Data Availability

Global WHEAT data source: https://www.kaggle.com/c/global-wheat-detection/data, accessed on 12 June 2020. Ground measurement data is available upon request due to privacy.
